# Comment on: “Aldehyde dehydrogenases contribute to skeletal muscle homeostasis in healthy, aging, and Duchenne muscular dystrophy patients” by Etienne et al.

**DOI:** 10.1002/jcsm.12609

**Published:** 2020-07-29

**Authors:** Juliane C. Campos, Che‐Hong Chen, Julio C.B. Ferreira

**Affiliations:** ^1^ University of Sao Paulo Institute of Biomedical Sciences Sao Paulo Brazil; ^2^ Department of Chemical and Systems Biology, School of Medicine Stanford University Stanford CA 94305 USA

As suggested by Etienne *et al*., exploiting how aldehyde metabolism contributes to skeletal myopathy is needed.^1^ These findings open up a new avenue for strategies targeting aldehyde dehydrogenases (ALDHs) as a regenerative approach. However, important questions should be addressed to pursue either ALDH‐enriched cell therapy or pharmacological ALDH activation as feasible interventions.

First, the use of Aldefluor to sort cells on the basis of ALDH activity should be interpreted with caution. ALDH family is composed of 19 functional isozymes. Each isozyme has a unique structural feature that renders specificity for certain type of aldehydes. For example, ALDH1A1 and ALDH2 have different catalytic tunnel conformation, which favours the oxidation of bulky (i.e. retinaldehyde) and small aldehydes (i.e. acetaldehyde), respectively.^2^ Using a single substrate (BODIPY aminoacetaldehyde, Aldefluor) to sort ALDH‐enriched cells favours certain types of ALDH isozymes, but not others, therefore limiting the comprehension of the heterogeneous nature of ALDH family. The use of this approach has an important value for general screening and sorting of samples with limited amount of tissue or cells, but it certainly underscores the ALDH contribution to skeletal muscle pathophysiology and regenerative capacity.

Second, the assay to sort ALDH‐enriched cells uses the molecule dimethylamino‐benzaldehyde (DEAB), as an inhibitor of ALDH (negative control). However, DEAB plays a dual role in ALDH biochemistry. It is a substrate for ALDH3A1, ALDH1A1, ALDH1A3, ALDH1B1, and ALDH5A1 isozymes (with different turnover rates) and a covalent inhibitor for ALDH1A2 and ALDH2.^3^ Therefore, the use of DEAB as negative control should be interpreted with caution if the main goal is to understand the individual contribution of ALDH isozymes to skeletal muscle regeneration. Future studies using selective substrates for individual ALDH isozymes are needed to unravel their contribution to myogenic progenitor cells proliferation, differentiation, and regenerative capacity.

Third, determining tissue distribution and transcript levels of different ALDH isozymes in healthy and Duchenne muscular dystrophy (DMD) patients as well as in myogenic cells in culture is a great accomplishment and suggests a possible association between some ALDH isozymes and regenerative phenotypes. However, these findings do not provide further insights regarding the contribution (causality) of any ALDH isozyme to skeletal muscle regeneration. Under this scenario, future studies using more selective genetic or pharmacological approaches for individual ALDH isozymes are required. There are some ALDH isozyme‐selective activators and inhibitors available that might be used to better understand their individual contribution to skeletal muscle pathophysiology.^4^, ^5^, ^6^, ^7^, ^8^, ^9^ For example, selective activation of ALDH2 counteracts cardiac degeneration induced by myocardial infarction.^10^, ^11^

Overall, Etienne *et al*. generated a very detailed anatomical distribution and mRNA transcript profile of different ALDH isozymes in skeletal muscle samples from DMD patients, dystrophic dogs, and non‐human primates. The next step will be the use of different substrates and selective modulators of ALDH isozymes. These approaches will allow a more detailed biochemical profile of ALDH isozymes as well as determine their individual contribution to skeletal muscle regeneration, therefore supporting the development of more selective strategies to tackle skeletal muscle diseases. It will be also interesting to understand whether such activators boost the skeletal muscle regenerative capacity of ALDH‐enriched stem cells. Finally, some genetic variations of ALDH isozymes (i.e. ALDH2 E504K mutation) affect over millions of people worldwide and render a severe enzymopathy.^4^, ^12^, ^13^, ^14^ However, the question whether such enzymopathies dampen skeletal muscle aldehyde metabolism and regenerative capacity remains to be answered.

## Conflict of interest

Che‐Hong Chen holds patents related to Alda‐1 activation of ALDH2 that are licensed to Foresee Pharmaceuticals. Juliane C. Campos and Julio C.B. Ferreira declare that they have no conflict of interest.
